# Effects of Different Tactical Formations on Positional Team Behaviors during Small Sided Games in Youth Soccer Players

**DOI:** 10.5114/jhk/194071

**Published:** 2024-12-19

**Authors:** Joaquín González-Rodenas, Jordi Ferrandis, Jorge Carril Valdó, Fernando Claver-Rabaz, Rafael Ballester, Alexander Gil-Arias

**Affiliations:** 1 Sport Sciences Research Centre, Rey Juan Carlos University, Fuenlabrada, Spain.; 2 Faculty of Physical Education & Sport Sciences, Catholic University of Valencia, Valencia, Spain.

**Keywords:** representative training, performance analysis, collective dynamics, game constraints, playing tactics

## Abstract

This study explored the impact of different tactical formations (TFs) on positional team behaviors in youth soccer during small sided games (SSGs). Eighteen U19 players participated in 7-a-side SSGs including goalkeepers, alternating between 2-3-1 and 3-1-2 TFs against a consistent opponent in the 3-3 TF. Positional data were collected with WIMU PRO GPS devices (Hudl, USA) to assess collective offensive and defensive dimensions that included teams´ width, length, height, the stretch index, and the surface area. The Mann-Whitney U test revealed that teams employing a 2-3-1 TF in offensive play exhibited increased height (p < 0.001; ES = 0.206), width (p = 0.006; ES = 0.113), and the surface area (p = 0.023; ES = 0.093) compared to the 3-1-2 TF. Conversely, defending with a 2-3-1 TF increased team height (p < 0.001; ES = 0.287) while decreasing length (p < 0.001; ES = 0.189), the surface area (p = 0.005; ES = 0.161), and the stretch index (p < 0.001; ES = 0.183) compared to the 3-1-2 TF. When attacking against a 2-3-1 TF, the offensive team experienced reduced height (p = 0.007; ES = 0.116) but an increased surface area (p < 0.001; ES = 0.241), width (p < 0.001; ES = 0.378) and the stretch index (p < 0.001; ES = 0.326) compared to the 3-1-2 TF. Finally, defending against a 2-3-1 TF resulted in decreased team length (p < 0.001; ES = 0.205), the surface area (p < 0.001; ES = 0.271) and the stretch index (p < 0.001; ES = 0.205) compared to defending against a 3-1-2 TF. Thus, coaches should acknowledge the significant role of TFs in modulating positional behaviors during SSGs, with relevant pedagogical implications for designing effective training sessions.

## Introduction

Soccer is a complex game where two teams have an interdependent tactical relationship due to their constant interaction and co-adaptation both in offensive and defensive moments throughout the match (Duarte et al., 2012). In this context, the collective behaviors of teams are constrained by the strategy and ability of the opposing team. This tactical understanding of the game requires soccer coaches to design representative practice tasks to potentiate the transfer from training to competition (Passos and Davids, 2015).

Small sided games (SSGs) have emerged as an ideal tool to create realistic training scenarios to optimize the holistic development of soccer players, encompassing technical, tactical and physical skills (Clemente et al., 2021; Sarmento et al., 2018), as well as optimizing the collective team behaviors (Davids et al., 2013; Ometto et al., 2018). In this sense, SSGs offer coaches the pedagogical possibility to manipulate different task constraints such as the field space, the number of players, the type of goals, etc., to expose players to specific game conditions and facilitate the emergence of functional movements patterns. In fact, the effect of modulating different task constraints on physical and physiological variables has been extensively documented in recent decades (Clemente et al., 2019; González-Rodenas et al., 2015; Praca et al., 2022; Rabano-Muñoz et al., 2023), helping fitness coaches to design more accurate training tasks to monitor the physical and physiological load of players (Clemente et al., 2014; Hammami et al., 2018; Prończuk et al., 2023, 2024; Skalski et al., 2024).

However, the investigation into the influence of task constraints on tactical behaviors is still undergoing a considerable increase in the last years (Ometto et al., 2018). Notably, the player positioning dynamics on the pitch has recently aroused as one of the key features to evaluate the functionality of players´ behavior as it captures the collective coordination according to the team principles of play (Coito et al., 2022). From this perspective, research has predominantly focused on the effects of manipulating the number of players (Aguiar et al., 2015; Vilar et al., 2014), the pitch size and configuration (Coutinho et al., 2018; Olthof et al., 2018), as well as task adjustments such as scoring methods (Travassos et al., 2014) or game rules (Castellano et al., 2016; Praca et al., 2021, 2022).

Nevertheless, despite that some studies in competition have highlighted significant effects of tactical formations (TFs) on technical and tactical performance (Aquino et al., 2020; Forcher et al., 2023; Memmert et al., 2019), the exploration of this task constraint within SSGs remains relatively sparse. In fact, TFs inherently establish a collective structure that defines the spatial arrangement and specific roles of players (González-Rodenas et al., 2023; Shaw and Glickman, 2019), what is key to define the interactions and synergies among various playing positions. For instance, Baptista et al. (2020) revealed that the number of midfielders used in TFs had a strong impact on the game dynamics and on space occupation during 7-a-side SSGs. That study observed that the 4-3-0 TF promoted players´ space exploration, the 4-1-2 TF promoted compactness and regularity of the team, while the 0-4-3 TF promoted team balance and adaptability to space coverage in relation to the opponent team.

Thus, there is a need to develop a broader understanding of the emergent collective behaviors upon manipulation of TFs in soccer SSGs. This understanding would assist coaches in designing specific team positional structures as part of the creation of representative learning environments for their players. Therefore, the aim of this study was to explore the impact of different tactical formations on positional team behaviors in youth soccer during 7-a-side SSGs.

## Methods

### Sample

A total of eighteen players (age = 17.9 ± 0.8 years; body mass = 69.2 ± 4.3 kg; body height = 178.0 ± 5.6 cm) participated in this study. Goalkeepers also participated in the protocol, but they were excluded from the data analysis. All players belonged to a sub-elite team located in Madrid that participated in a top-level competition according to their age category. The key inclusion criteria were to be an official player of the team and to be fully prepared physically to sustain the demands of SSGs. Thus, tryout players and players undergoing a preparation process after injury were excluded from the study. The investigation was approved by the institutional ethics committee of the Rey Juan Carlos University (protocol code: 1505202322823; approval date: 15 November 2023) and followed the ethical standards for study in humans as outlined in the Declaration of Helsinki.

### Experimental Approach

A cross sectional design was used to investigate the effects of attacking and defending with different TFs during SSGs in training sessions. Before the beginning of the study, the coaching staff, in conjunction with the research team, created two competitively balanced teams (A and B) that were kept stable throughout the intervention. In this sense, players from the whole squad were selected for each team based on their specific playing positions and their technical-tactical skills. The selection of playing positions was based on the technical and tactical profile of players, considering their most usual positioning during the season in competition and training sessions.

Additionally, one training session of familiarization was performed for coaches and players to experience the SSGs and the TF established in the study.

Table 1 shows the main characteristics of SSGs implemented in this study. These SGGs consisted of 7-a-side games (including goalkeepers) played in a field of 65 m long and 50 m wide (232 m^2^ per player) that reflected a representative learning design where the game constraints were similar to the real competition, but with the reduced number of players and pitch size (Passos and Davids, 2015). For that purpose, official soccer rules were applied including the off- side rule, with the only exception of restarts that all were taken as goal kicks.

**Table 1:** Design of the small sided games.

Task constraints	Description
**Player number (including goalkeepers)**	7v7
**Floaters**	No floaters
**Area per player (m^2^)**	232 m^2^
**Time (work: passive recovery)**	(5:2 minutes)
**Tactical objective**	**Offensive:** to create goal scoring opportunities by penetrating the opposing team defensive structure. **Defensive:** to form a compact block to prevent opposing penetration.
**Coach´s feedback**	No direct instructions
**Rules**	Official soccer rules including off-side. The only exception is that all the restarts are taken as goal kicks (no throw ins, corner kicks or free kicks). When a goal-kick is taken, the opposing team has to start in a medium defensive block
**Type of finishing**	7-a-side goals
**Tactical formations**	2-3-1 vs. 3-3 3-1-2 vs. 3-3

Furthermore, both teams shared the tactical objectives of disordering the opposing team to create goal scoring opportunities and preventing the penetration by trying to maintain a compact defensive block.

**Figure 1:**
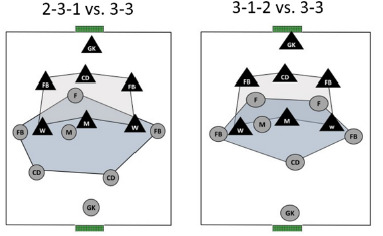
Representation of the SSGs implemented in the study.

During SSGs, teams A and B implemented three different TFs to represent a tactical context where TFs 2-3-1 and 3-1-2 were always confronted with a 3-3 TF to have two different TFs playing versus a similar opposition (Figure 1).

SSGs were performed once per week for a period of three weeks as part of the regular training sessions of the U19 team. In total, players performed eighteen games of five minutes with two minutes of rest between bouts, where different TFs (2-3-1, 3-1-2 and 3-3) were randomly assigned to each team according to the study design (Table 2). In this design, both teams alternated tactical formations during three or two subsequent periods during the same day, in such a way that always one team played a 3-3 TF and the other team formed a 2-3-1 or a 3-1-2 TF. In this way, both teams played with the three different TFs during the intervention.

**Table 2:** Experimental design that shows the tactical formations employed by each team in each period.

Period (5 min)	SESSION 1		SESSION 2		SESSION 3	
	Team A	Team B	Team A	Team B	Team A	Team B
**1**	2-3-1	3-3	3-3	3-1-2	3-3	2-3-1
**2**	2-3-1	3-3	3-3	3-1-2	3-3	2-3-1
**3**	2-3-1	3-3	2-3-1	3-3	3-3	2-3-1
**4**	3-3	3-1-2	2-3-1	3-3	3-1-2	3-3
**5**	3-3	3-1-2	3-3	2-3-1	3-1-2	3-3
**6**	3-3	3-1-2	3-3	2-3-1	3-1-2	3-3

Prior to each bout, players were informed about the TF that their team had to form in a tactical board by their coach, as well as the playing position that each player had to occupy in that formation. However, no specific tactical instructions were provided apart from the objectives of creating and preventing goal scoring opportunities and goals. In this context, players were free to make decisions and actions during SSGs, considering the tactical role assigned to their playing position. Furthermore, coaches did not provide direct or indirect instructions to players, in order to not to influence their decisions and actions during SSGs.

SGGs were performed following a standardized warm-up protocol of two phases of five minutes each. Initially, players engaged in dynamic mobility exercises encompassing activities involving jumps, dynamic stretching routines, and directional changes. Subsequently, the second phase centered on a passing exercise designed to emphasize both passing proficiency and dribbling while running. All the training sessions took place during the same hours (7.00 p.m.), on the same artificial turf surface.

***Procedures***

Players’ movements during SSGs were recorded using WIMU PRO™ GPS devices (Hudl, USA) operating at a sampling frequency of 10 Hz. A total of 9049 collective positions were registered through the experimental design. These devices are a reliable technology to monitor soccer players’ movements (Bastida-Castillo et al., 2019). For the collection of data, the research team set up the devices next to the training field in a flat and clear space to ensure the optimal signal, following the manufacturer´s recommendations. Prior to the training session, GPS devices were inserted in a purpose-built vest (anatomically adjusted to each participant) that players wore, in such a way that the device was on the upper part of the players' back.

With this system, five tactical variables related to the collective positioning of teams (Low et al., 2020; Rico-González et al., 2022) during offensive and defensive phases were calculated using SPRO software (WIMU, Hudl, USA):

- Team height: Mean team defensive depth per match, understood as the distance in meters (m) between the furthest back player and the goal being defended
- Team width: Mean team width per match, understood as the distance in meters (m) between the two players furthest-apart across the width of the pitch
- Team length: Mean team length per match, understood as the distance in meters (m) between the two players furthest-apart along the length of the pitch
- Stretch index: Mean distance from each player position to the geometrical center of the corresponding team
- Team surface area: Mean team surface area per match, understood as total square meters (m^2^) of a polygon described by players as its vertex point and calculated using the convex hull calculation.

These tactical variables are useful to evaluate the spatial exploration and inter-player distances of teams during SSGs, what allows to evaluate and compare the collective coordination and behavior of teams. The analysis of these variables has been used in previous investigations both in real competition and SSGs (Baptista et al., 2020; Low et al., 2020, 2022; Ometto et al., 2018; Praca et al., 2021).

### Statistical Analysis

The data were transferred from SPRO software (Hudl, USA) to a Microsoft Excel database, where it was organized and exported to SPSS (IBM, version 27.0, USA). A Kolmogorov-Smirnov test indicated that the data were nonparametric. Therefore, medians and interquartile ranges (IQR) were reported, and the Mann-Whitney U test was conducted for the comparison of the different TFs. Finally, Cohen’s *d* was calculated to show the magnitude of effect sizes (ESs) associated with the dependent variables for each population (Cohen, 1988). ESs were evaluated using the approach of Cohen (1992) where 0–0.2 represents a trivial effect, >0.2–0.5 represents a small effect, >0.5–0.8 a medium effect and >0.8 a large effect. Data were presented in tables and boxplot graphs to assess and compare the shape, the central tendency and variability of the sample. The significance level was set at *p* < 0.050.

## Results

### Attacking with a 2-3-1 or a 3-1-2 TF

Table 3 shows that attacking with a 2-3-1 structure provided higher team’s height on the field (*p* < 0.001; ES = 0.206), as well as more team’s width (*p* = 0.006; ES = 0.113) than attacking with a 3-1-2 structure, although no differences between TFs were found in relation to the team’s length and the stretch index.

**Table 3:** Comparative analysis of collective tactical variables according to the TF and the tactical moment.

Tactical moment	Collective tactical variable	Tactical formation		*p*	ES
		2-3-1 Median (IQR)	3-1-2 Median (IQR)		
**Offensive**	Height (m)	29.6 (18.7–35.3)	27.2 (13.7–33.6)	< 0.001	0.206
	Width (m)	32.8 (26.5–38.2)	31.5 (25.1–37.7)	0.006	0.113
	Length (m)	21.0 (17.6–25.4)	21.3 (16.7–27.2)	0.461	0.030
	Stretch index (m)	12.8 (11.5–14.1)	12.7 (11.4–14.4)	0.788	0.011
**Defensive**	Height (m)	16.5 (10.4–26.5)	14.4 (8.3–21.3)	< 0.001	0.287
	Width (m)	26.2 (22.3–29.6)	25.5 (21.2–30.8)	0.538	0.027
	Length (m)	20.1 (14.8–25.5)	21.6 (15.9–27.3)	< 0.001	0.189
	Stretch index (m)	11.1 (9.1–12.9)	11.7 (9.5–13.4)	< 0.001	0.183

**Figure 2:**
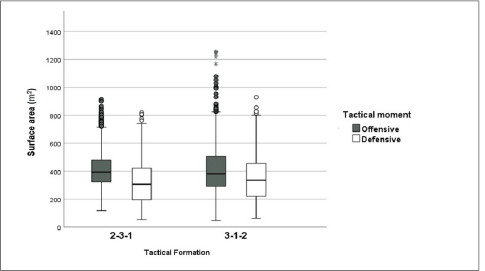
Team surface area according to the tactical formation and the tactical moment in 7-a-side SSGs. The box indicates the 25^th^ and 75^th^ quartiles and the central line is the median. The ends of the whiskers are the 2.5% and 97.5% values. Values outside the range of the whiskers are extreme values.

Furthermore, Figure 2 depicts that the 2-3-1 TF registered a larger team surface area (median = 394.8 m^2^; IQR: 322.5–481.0 m^2^) compared to the 3-1-2 formation (median = 380.9 m^2^; IQR: 292.4–505.5 m^2^) (*p* = 0.023) although the effect size was trivial (ES = 0.093).

### Defending with a 2-3-1 or a 3-1-2 TF

As for the defensive positioning, the 2-3-1 structure registered higher team’s height (*p* < 0.001; ES = 0.287) but lower length (*p* < 0.001; ES = 0.189) and the stretch index than defending with a 3-1-2 structure. Besides this, the 2-3-1 structure had a smaller team surface area (median = 305.5 m^2^; IQR: 195.9–421.9 m^2^) than the 3-1-2 TF (median = 335.7 m^2^; IQR: 219.2–454.7 m^2^) (*p* = 0.005; ES: 0.161).

### Attacking vs. a 2-3-1 or a 3-1-2 TF

**Figure 3:**
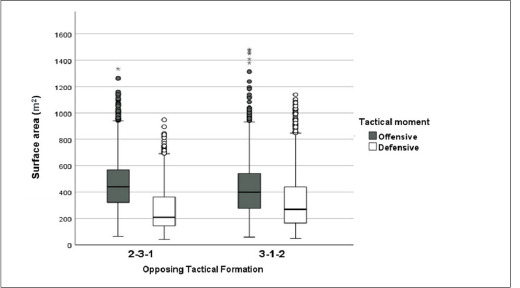
Team surface area according to the opposing tactical formation and the tactical moment in 7-a-side SSGs. The box indicates the 25^th^ and 75^th^ quartiles and the central line is the median. The ends of the whiskers are the 2.5% and 97.5% values. Values outside the range of the whiskers are extreme values.

When building up an attack against a 2-3-1 TF, the offensive team exhibited lower team´s height but more team´s width and the stretch index than attacking against a 3-1-2 TF (Table 4). In addition, attacking against a 2-3-1 TF increased the team´s surface area in comparison with a defensive 3-1-2 structure (*p* < 0.001; ES = 0.241) (Figure 3).

**Table 4:** Comparative analysis of collective tactical variables according to the opposing TF and the tactical moment.

Tactical moment	Collective tactical variable	Opposing tactical formation		*p*	ES
		2-3-1 Median (IQR)	3-1-2 Median (IQR)		
**Offensive**	Height (m)	20.3 (11.6–30.1)	21.6 (14.1–29.4)	0.007	0.116
	Width (m)	34.2 (28.1–38.9)	30.0 (24.9–36.3)	<0.001	0.378
	Length (m)	22.9 (18.6–27.3)	22.5 (17.3–28.1)	0.800	0.011
	Stretch index (m)	13.8 (11.8–15.6)	12.7 (10.5–14.6)	<0.001	0.326
**Defensive**	Height (m)	11.8 (7.6–18.2)	11.0 (7.4–19.8)	0.765	0.012
	Width (m)	25.4 (21.3–30.2)	25.1 (21.7–29.3)	0.195	0.053
	Length (m)	14.2 (10.3–20.9)	18.1 (12.1–26.8)	<0.001	0.405
	Stretch index (m)	9.9 (8.3–12.4)	10.7 (8.7–13.5)	<0.001	0.205

### Defending vs. a 2-3-1 or a 3-1-2 TF

Defensively, when the opposing team attacked with a 2-3-1 structure, the defensive team registered lower length (*p* < 0.001; ES = 0.405) and the stretch index (*p* < 0.001; ES = 0.205) than when defending against a 3-1-2 TF (Table 4). Additionally, defending against a 2-3-1 offensive structure increased the team´s surface area (median = 209.8 m^2^; IQR; 145.1–364.0 m^2^) in comparison with defending against a 3-1-2 team structure (median = 269.4 m^2^; IQR: 163.7–438.9 m^2^) (*p* < 0.001) with a small effect size (ES = 0.271) (Figure 3).

## Discussion

The aim of this study was to explore the impact of different TFs on positional team behaviors in youth soccer during 7-a-side SSGs. Our investigation revealed that offensive and defensive collective dynamics of teams significantly varied depending on the TF employed for both teams. This study provides novel pedagogical insights to understand the design of representative learning tasks (Passos and Davids, 2015) during training sessions from a nonlinear dynamical perspective, that allow us to view collective tactical behaviors as emergent, dynamic actions performed based on match interactions (Duarte et al., 2012; Low et al., 2020; Silva et al., 2016).

In this regard, playing with a 2-3-1 TF facilitated a slightly more expansive offensive formation compared to the 3-1-2 TF. This divergence may be induced by the fact that the 2-3-1 TF has two central defenders that are constantly situated at the back of the TF. This creates a more balanced structure during the attacks, allowing greater freedom for wingers to widen their positioning and advance into offensive areas. According to our results, the 2-3-1 structure had a more stable positioning and a surface area when attacking, while the 3-1-2 structure had more variability in the collective behaviour with more extreme values especially regarding the team surface area. This higher variability coupled with a less expansive offensive formation in the 3-1-2 structure might be attributed to the solitary central defender, influencing wingers to assume a more defensive role, thereby maintaining numerical balance during attacks and potentially limiting team width compared to the 2-3-1 TF.

In terms of defensive positioning, the 2-3-1 TF demostrated more height on the field, as well as a more compact structure with less team length, a lower surface area and the stretch index, compared to the 3-1-2 structure. In this sense, the inclusion of three players in the midfield line alongside the forward within the 2-3-1 TF could facilitate a higher defensive line, positioning four players in advanced positions during defensive phases, contrary to the 3-1-2 structure which featured fewer players in the midfield and forward lines. Furthermore, the 2-3-1 TF has a very symmetrical and balanced spatial organization so that players’ distances across different lines are potentially smaller than in the 3-1-2 TF, what allows players to be compact and closer to their teammates when defending.

Our study not only found that the 2-3-1 and 3-1-2 TFs modulated the collective dynamics of implementing teams, but also constrained the collective behaviour of the opposing team in defensive and offensive moments. In this regard, the opposing teams (forming a 3-3 TF), exhibited reduced team height but an increased surface area, width and the stretch index when attacking against a 2-3-1 TF, in comparison with attacking against a 3-1-2 TF. In this regard, the defensive structure of the 2-3-1 TF compelled opposing teams to adopt a lower offensive position while widening their stance to disrupt the 2-3-1 TF's compactness. Conversely, the 3-1-2 defensive configuration encouraged higher offensive positioning, despite maintaining a less expansive offensive structure.

Concerning the defensive moment against a 2-3-1 TF, it resulted in decreased team length, the surface area, and the stretch index, while defending against a 3-1-2 amplified defensive length and the surface area. In this sense, the more expansive structure of the 2-3-1 TF in the offensive moment would provoke the necessity for the defending team to get compact to prevent the offensive penetration. On the other hand, defending against a 3-1-2 TF seems to increase the defensive length and the surface area. This may be due to the more variable and less expansive offensive positioning of the 3-1-2 TF, that allows the defensive team to have more distance between players, especially in length.

These findings show the emergence of collective dynamics due to the interaction and co-adaptation of different TFs during representative SGGs in youth soccer players. Previous research had explored the influence of TFs on collective dynamics under real match conditions. For instance, Low et al. (2022) observed that the underlying mechanisms in players’ collective movements and passing showed that the 5-3-2 structure was more conservatively defensive than the 4-4-2 TF because of higher compactness. Also, Memmert et al. (2019) did not observe tactical differences between the 4-2-3-1 and the 3-5-2 structure in effective playing space or team separateness, although the 3-5-2 TF showed a higher player length per width ratio than the 3-5-2 TF.

However, according to our knowledge, this is the first study to specifically evaluate the influence of these TFs on collective dynamics in SSGs, what makes it impossible to compare our results with similar research studies. In this regard, Baptista et al. (2020) compared the effects of forming three different TFs and playing positions: 4-3-0 (4 defenders and 3 midfielders), 4-1-2 (4 defenders, 1 midfielder and 2 forwards) and 0-4-3 (4 midfielders and 3 forwards) on tactical behavior. That study showed that the team constituted by defenders, midfielders, and forwards (4-1-2) promoted more compactness, the team constituted by defenders and midfielders (4-3-0) promoted more space exploration with higher values of team length, width and the surface area, while the team of midfielders and forwards (0-4-3) showed a more balanced behavior. Another study (Santos et al., 2023) observed that different behaviors emerged from the manipulation of the number of creative players in the opposing team, highlighting how the inclusion of specific players in each team can constrain the collective dynamics of teams. Complementary to our results, those findings show the importance for coaches to consider not only the TFs, but also the skill level and characteristics of players when designing SSGs, so that different tactical behaviors can emerge depending on the teams and players’ confrontation.

This study has some limitations that need to be addressed. Firstly, our research was based on collective variables, what does not show individual tactical behaviors or comparison between different playing positions. Secondly, our analysis was based on positional data, what shows the collective movements of teams, but it does not describe the technical and tactical actions performed during SSGs. Finally, the current study was carried out with a small sample size of sub-elite youth male players and the results should not be extrapolated to other categories or to women´s soccer.

Nevertheless, our investigation has important practical implications and conclusions. In this sense, soccer coaches should consider the impact of different TFs on collective dynamics when designing SGGs in youth soccer players. Specifically, the utilization of a 2-3-1 TF fosters a more expansive offensive setup and a more compact, elevated defensive structure compared to the 3-1-2 TF which exhibits greater variability in spatial exploration. Furthermore, the 2-3-1 TF prompts the opposing team to adopt a more compact defensive structure while assuming a more expansive, lower offensive formation, distinct from encounters involving the 3-1-2 TF. This information is highly relevant for coaches due to its applied value, since knowing the offensive and defensive collective dynamics of each TF may help them to better design training scenarios to develop their game model.
